# Acute abdomen due to torsion of the wandering spleen in a patient with Marfan Syndrome

**DOI:** 10.1186/1749-7922-8-30

**Published:** 2013-08-05

**Authors:** Laura Leci-Tahiri, Afrim Tahiri, Rifat Bajrami, Mehmet Maxhuni

**Affiliations:** 1 Clinic of Surgery, University Clinical Center of Kosova, Prishtina, Kosova

## Abstract

Wandering spleen is a very rare defect characterized by the absence or weakness of one or more of the ligaments that hold the spleen in its normal position in the upper left abdomen. Patient symptomatology is variable and ranges from mere feeling of an abdominal lump to sudden abdominal pain due to infarction. Patients may have subacute to chronic abdominal or gastrointestinal complaints. Because of nonspecific symptoms, clinical diagnosis can be difficult; hence, imaging plays an important role. A major complication is splenic torsion, which is the cause of acute abdomen. We present a case of acute abdominal pain due to torsion of wandering spleen in a patient with Marfan Syndrome, valvular heart disease, and vertebral anomalies. Preoperative diagnosis was made on the basis of ultrasonography and computed tomography, which was later confirmed on surgery, and treated successfully.

## Case presentation

**Figure 1:**
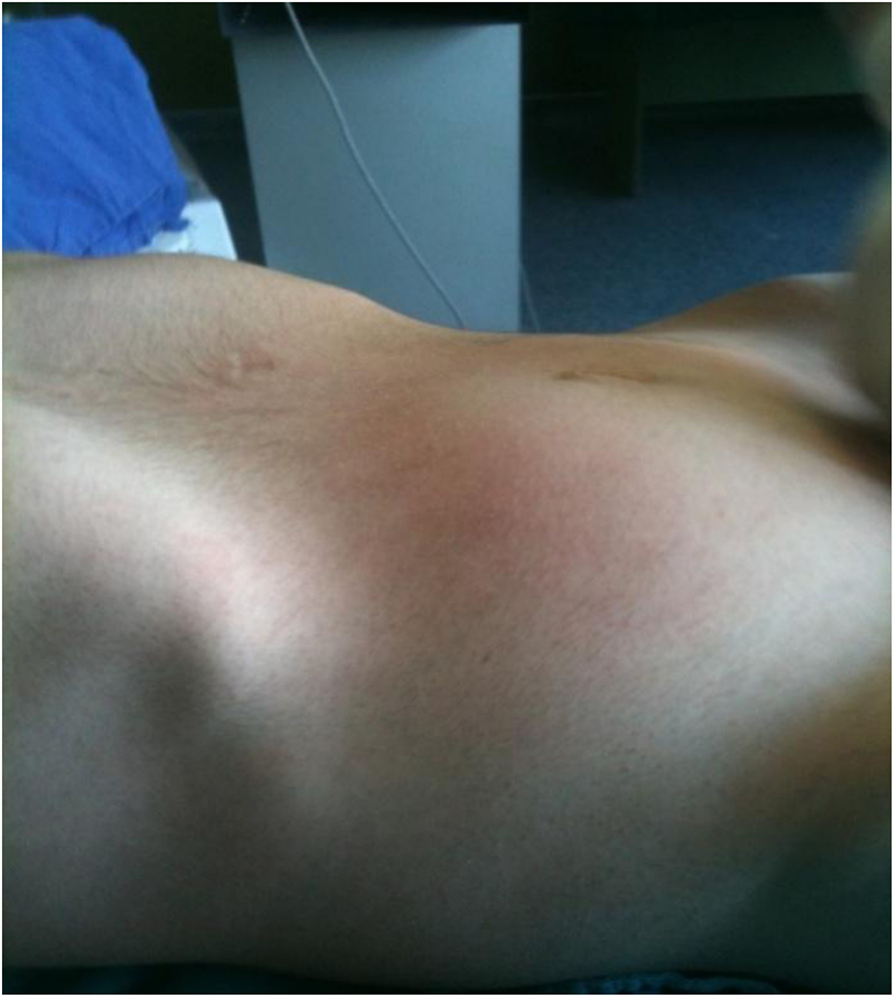
Tumor in the right lower abdomen.

A 36-year-old Albanian man presented to Emergency Unit with complaints of abdominal pain, two-week history of constipation, and a tumor in the right lower abdomen (Figure 1).

The patient presented with features of Marfan syndrome: increased height, arachnodactyly, long limbs, contractures of the hand, pectus excavatum, genu recurvatum, and scoliosis. He had undergone mitral valve implantation 15 years previously, and had been treated with oral anticoagulants.

At admission, the patient was afebrile, pale, rundown, and fully conscious. His left lower extremity was oedematous under the knee. Abdomen was soft on palpation with a 20×9 cm mass palpable in the right hypogastric region.

Doppler examination of the lower extremity veins showed thrombosis of the left popliteal and left tibialis posterior vein. A vascular surgeon was consulted, and heparin with a high molecular weight, 7500 UI, was administered every 6 hours intravenously.

Due to lung problems, a pulmonologist was further consulted, who found pleuropneumonia in the left lung. The patient suffered from arterial hypertension and chronic cardiomyopathy.

Laboratory investigations showed mild anaemia and leucocytosis. Tumor markers were checked but were all within normal limits.

### Ultrasound of the abdomen

Absence of the spleen in its normal position in the left hypochondrium, and presence of tumor mass in the right fossa inguinalis. Other organs of the abdomen were normal.

### Magnetic resonance imaging of the abdomen and pelvis

Absence of the spleen in the normal location. The spleen was seen in the lower right hemiabdomen, enlarged, with the size of 18.7×8.5×20.8 mm and sacral meningocoele.

### CT angiography of abdominal vessels

**Figure 2:**
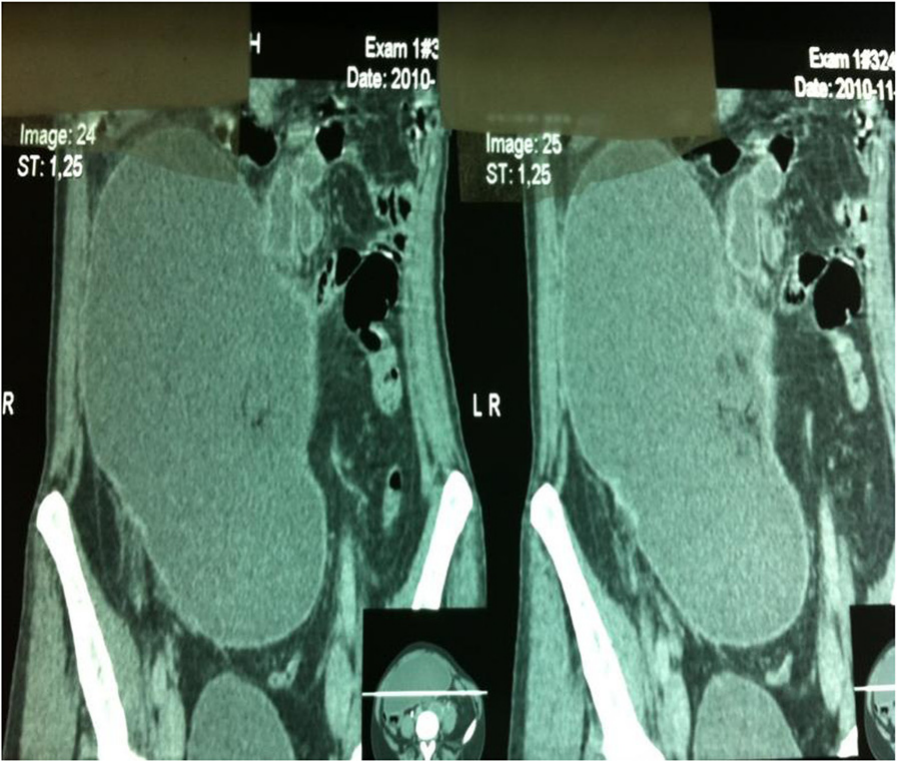
Anteroposterior Angio-CT showing enlarged spleen in lower right hemiabdomen.

**Figure 3:**
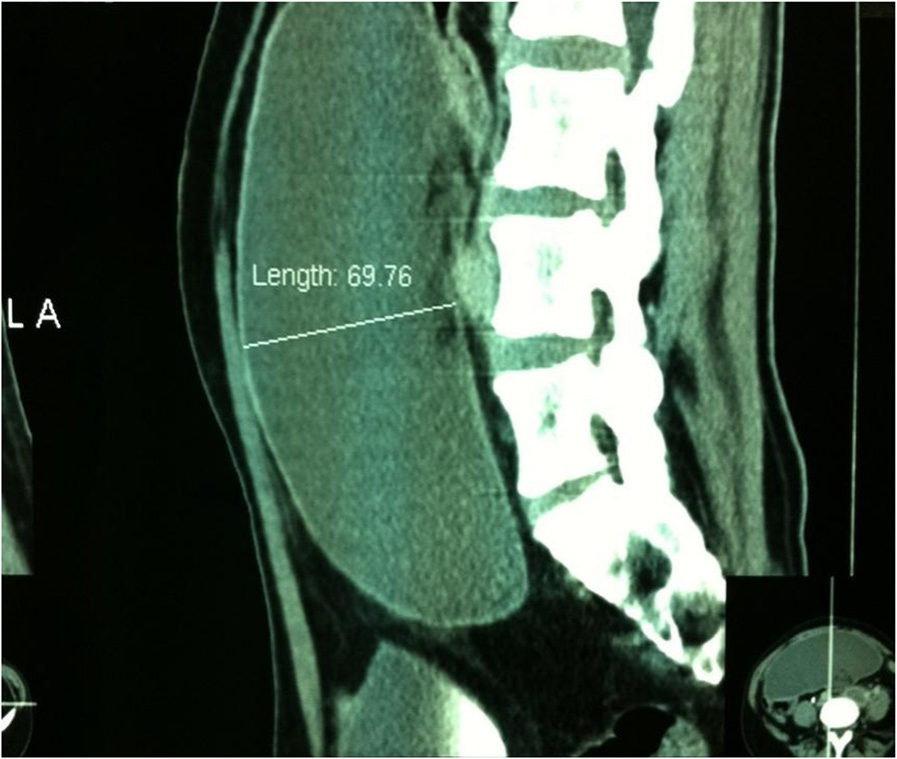
Sagital Angio-CT showing size of spleen.

Splenic artery was divided by pancreatic artery, which was forwarded to the tail of pancreas giving it a “whorled appearance”, and from this level splenic vessels were thrombosed. Pancreas was moved forward without obvious radiological changes (Figures 2 and 3).

**Figure 4:**
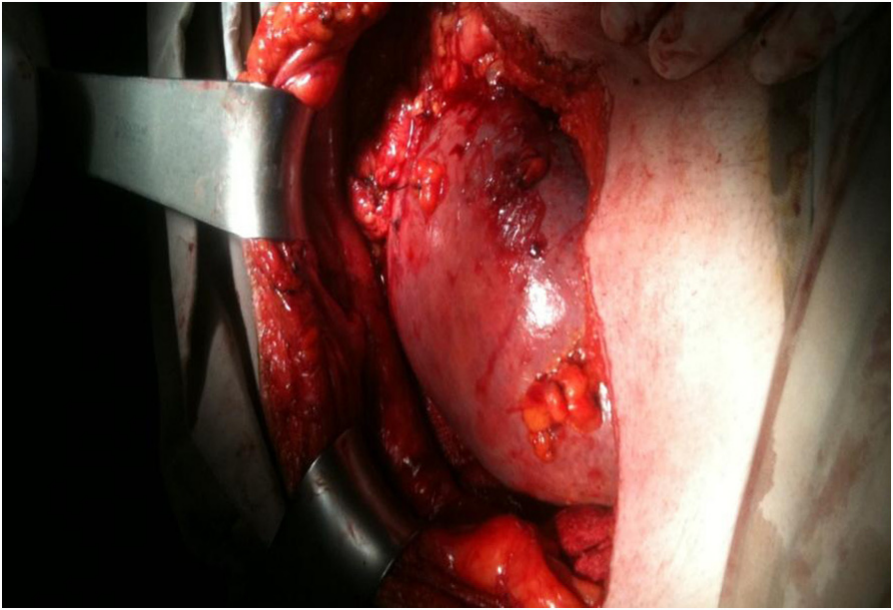
Huge spleen in right pelvic area.

**Figure 5:**
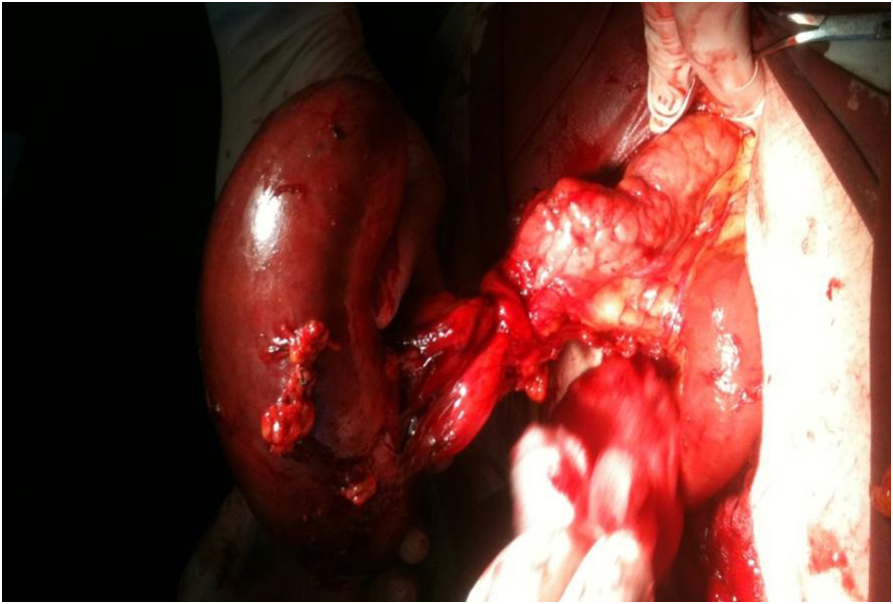
“Whirl sign” in the area of the splenic vascular pedicle, indicative of torsion.

Operative findings revealed a huge spleen in the pelvic area with torsion of the vascular pedicle starting at the tail of the pancreas (Figure 4). The characteristic “whirl sign” can be seen in the area of the splenic vascular pedicle, indicative of torsion (Figure 5). Other internal organs were normal.

**Figure 6:**
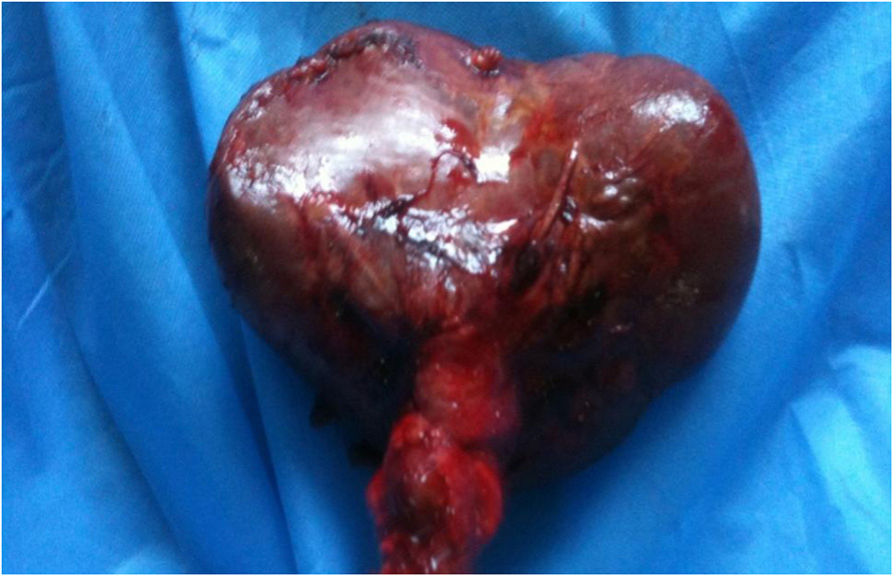
Spleen with diffuse hemorrhagic and ischemic infarcts.

A total splenectomy was performed, as the organ appeared congested, it was likely infarcted and not likely to be salvageable (Figure 6).

The patient recovered well after the operation. Antibiotics, analgesics, plasma, blood, low molecular weight heparin, vitamins and triple vaccination (against pneumococcus, hemophilus influenza, and meningococcus) were given.

He was discharged on oral anticoagulants because of heart disease.

Histology revealed acute thrombotic changes in arteries and veins of the splenic hilum, with diffuse hemorrhagic and ischaemic infarcts of the spleen.

## Discussion

Wandering spleen is an uncommon clinical entity, which rarely affects children and adolescents. Discussion in the literature has been limited to case reports and small case series [1]. The condition is not hereditary.

Congenital wandering spleen is a very rare randomly distributed birth defect characterized by the absence or weakness of one or more of the ligaments that hold the spleen in its normal position in the upper left abdomen. Instead of ligaments, the spleen is attached by a stalk-like tissue supplied with blood vessels (vascular pedicle). If the pedicle is twisted in the course of the movement of the spleen, the blood supply may be interrupted or blocked (ischaemia) to the point of severe damage to the blood vessels (infarction). Because there is little or nothing to hold it in place, the spleen “wanders” in the lower abdomen or pelvis where it may be mistaken for an unidentified abdominal mass.

“Acquired” wandering spleen may occur during adulthood due to injuries or other underlying conditions that may weaken the ligaments that hold the spleen in its normal position (connective tissue disease or multiparity) [2,3].

Van Horne, a Dutch physician, is credited with describing this condition in 1667 after performing an autopsy. In 1875, Martin, a German obstetrician, performed the first splenectomy for a wandering spleen [4,5]. Ten years later, splenopexy was described and considered superior to splenectomy, a differential preference that has changed several times over the years. Since Van Horne’s discovery, approximately 400 cases of wandering spleen have been reported worldwide. It is a rare entity accounting for less than 0.25% of splenectomies [6]. Twenty one cases of wandering spleen, including our present case, have been reported in the English literature during the past decade (Table 1). The majority of patients are female, in second and third decade of life. Computed tomography is the imaging method of choice for diagnosing wandering spleen. The usual location of wandering spleen is pelvis and left iliac fossae. We couldn’t find in literature the location in right iliac fossa, as our case showed. Abdominal pain, intestinal obstruction, nausea, vomiting, fever, and a lump in the abdomen or the pelvis are the common symptoms in all reported cases. Splenectomy is performed in most cases.

**Table 1:** The characteristics of the reported cases of wandering spleen

**Case**	**Age**	**Gender**	**Diagnostic modality**	**Spleen location**	**Type of surgery performed**	**Reference**
1	26	F	CT	Hypogastric region	Splenectomy	Pan Afr Med J 2012
2	27	F	US, CT	Left lower quadrant	Splenopexy	Saudi J Gastroenterol 2010
3	28	F	CT	Left lower quadrant	Splenopexy	Case Rep Surg 2013
4	44	M	CT	Lower pelvis	Splenectomy	N Am J Med Sci 2011
5	20	F	CT	Right upper quadrant	Splenopexy	JSLS 2008
6	19	F	Doppler, GI endoscopy	Left iliac fossa	Splenopexy	JSLS 2007
7	41	F	CT	Left lower quadrant	Splenectomy	JSLS 2012
8	21	F	CT	Intrathoracal	Splenopexy	J Blood Med 2011
9	9	F	CT	Periumbilical	Splenectomy	Br J Radiol 2010
10	15	M	CT	Left iliac fossa	Splenectomy	Cases J 2008
11	64	M	CT	Left hemothorax	Splenectomy	BMC Gastroenterol 2006
12	28	F	CT	Pelvis	Splenectomy	Am J Surg 2008
13	21	F	US, CT	Pelvis	Splenectomy	Hong Kong Med J 2012
14	9	F	CT	Pelvis	Splenectomy	PediatrEmerg Care 2003
15	4	F	US, CT	Left lower quadrant	Splenectomy	ActaRadiol 2011
16	4	F	CT	Left hemothorax	Splenopexy	AJR 2012
17	28	F	US,CT	Right upper quadrant	Splenectomy	Singapore Med J 2007
18	30	F	CT	Left lower quadrant	Splenectomy	BratislLekListy 2009
19	19	F	CT	Pelvis	Splenectomy	BratislLekListy 2009
20	16	F	US	Pelvis	Splenopexy	SA FamPract 2010
21	36	M	CT	Right iliac fossa	Splenectomy	Present study

Discussion in the literature is limited, especially in cases with Marfan Syndrome and valvular heart disease. We have found only one case with wandering spleen in a child with Marfan Syndrome [7].

Marfan syndrome is caused by a defect, or mutation, in the gene that determines the structure of fibrillin-1, a protein that is an important part of connective tissue. It is an inherited disorder of the connective tissue that affects major organ systems of the body: the heart and circulatory system, thebones and muscles, and the eyes. A person with Marfan syndrome is born with the disorder, even though it may not be diagnosed until later in life [7].

As it is a generalized connective tissue disorder, congenital laxity of the primary ligamentous attachments of the spleen might predispose to splenic hypermobility and hence torsion in childhood, in contrast to the more common acquired form of splenic torsion seen in multiparous females that is believed to be caused by laxity of these ligaments owing to hormonal changes and multiparity [7-9].

Symptoms of wandering spleen are those typically associated with an abnormal size of the spleen (splenomegaly) or the unusual position of the spleen in the abdomen [9,10].

Patients maybe asymptomatic or may present with acute abdominal pain. The common clinical presentation is abdominal mass with pain. It may occur in people of all ages with a predilection for male under 10 years of age and for female patients in older age groups, being most common in multiparous women. Under the age of 10 the sex distribution is even, whereas over 10 years of age, females out number males by seven to one. A study involving 66 children under 10 years showed that 50% of wandering spleens were lost through acute ischaemia [7,9,11].

Splenic torsion is usually clockwise. Complications of splenic torsion include: gangrene, abscess formation, local peritonitis, intestinal obstruction and necrosis of the pancreatic tail, which can lead to recurrent acute pancreatitis [6,12,13].

Splenopexy is the treatment of choice for a noninfarcted wandering spleen. One small case study in 2004 demonstrated successful laparoscopic splenopexy using a Vicryl mesh bag. Splenic preservation in cases of wandering spleen without rupture or infarction avoids the risk of overwhelming postsplenectomy sepsis, and a laparoscopic approach allows for shorter hospital length-of-stay and decreased postoperative pain [12,14,15].

Splenectomy should be done only when there is no evidence of splenic blood flow after detorsion of the spleen. In our patient, because of the intraoperative findings of splenic infarction, splenectomy was performed [12,16].

## Conclusion

The possible diagnosis of wandering spleen should be kept in mind when CT shows the spleen to be absent from its usual position and a mass is found elsewhere in the abdomen or pelvis. Abdominal ultrasonography (with or without Doppler) and CT are useful investigative tools. Early intervention is necessary to reduce the risk of splenic infarction and other complications. An awareness of the condition together with the use of appropriate medical imaging can lead to the correct diagnosis.

### Consent

Written informed consent was obtained from the patient for publication of this case report and any accompanying images. A copy of the written consent is available for review by the Editor-in-Chief of this journal.

## Competing interests

The authors declare that they have no competing interests.

## Authors’ contributions

AT and RB performed the surgery, supervised the patient’s care, drafted the manuscript, and approved the version submitted for publication. LT and MM assisted with patient care and have been involved in drafting the manuscript. AT, LT and MM has been involved in drafting and revising the manuscript. All authors read and approved the final manuscript.
